# Risk, Transformation and Adaptation: Ideas for Reframing Approaches to Disaster Risk Reduction

**DOI:** 10.3390/ijerph16142594

**Published:** 2019-07-20

**Authors:** Douglas Paton, Petra Buergelt

**Affiliations:** 1 College of Health and Human Sciences, Charles Darwin University, Darwin, NT 0810, Australia; 2 Faculty of Health, University of Canberra, 11 Kirinari St, Bruce, ACT 2617, Australia

**Keywords:** transformation, adaptive capacity, risk, learning, scenario planning, disaster risk reduction, disaster recovery

## Abstract

Recognition of projected increases in exposure to large-scale hazard events over the coming decades has identified a need to develop how disaster risk reduction and recovery are conceptualized and enacted. This paper discusses some strategies for pursing this goal in both disaster recovery and preparedness settings. The approaches discussed include understanding how communities learn from their hazardous experiences and transform these lessons into beliefs, relationships and capabilities that build future adaptive capacity. The paper draws on examples of transformative learning that illustrate how people can make fundamental shifts in how they think about, prepare for and respond to environmental challenge and change. Regarding transformation in pre-event settings, the paper first discusses why the addition of transformative strategies to disaster risk reduction programs is required. These include a need for rethinking socio-environmental relationships, increasing risk acceptance in the context of evolving hazardscapes, and countering beliefs regarding not preparing. The paper then offers strategies for motivating transformation and consolidating the outcomes of transformation in pre-event disaster risk reduction (DRR) strategies. A preliminary model that could inform the development of research questions on the development of transformative outcomes and their consolidation in enduring adaptive processes is presented.

## 1. Introduction

Worldwide, people’s exposure to environmental hazards and disasters will increase over the coming decades. This reflects the influence of factors ranging from population growth to infrastructure development to urban spread to increases in the frequency and intensity of hazards from, for example, storm, hurricane (cyclone/typhoon) and wildfire hazards. When disaster does strike, affected societies and citizens experience circumstances (e.g., meeting survival needs, adapting to loss of infrastructure and disruption to livelihood issues etc.) that differ substantially from anything they would encounter in everyday life, and often for prolonged periods of time [1]. Consequently, people will benefit from being prepared in ways that increase their capacity to respond to disaster, and to do so independently of societal support. However, despite the time and resources allocated to communicating risk, advising citizens about their need for preparedness and providing information about how to prepare, levels of adoption of preparedness measures remain low [2,3,4,5,6,7]. This lack of preparedness increases societal losses when disaster strikes and prolongs recovery times in affected communities. In an era in which disasters are projected to increase in frequency and severity, a need to proactively develop response and recovery capability has become a disaster risk reduction (DRR) imperative [8].

The authors introduced in the previous paragraph offered suggestions for the low levels of preparedness uptake. A key issue identified concerns the fact that citizens think about preparedness in ways that can differ substantially from their professional risk management counterparts. This draws attention to a need to understand how people think about preparedness and how these beliefs are or are not converted into relevant knowledge and action. s. This underpins arguments for a need to rethink how preparedness strategies are conceptualized and implemented. One way of doing so involves facilitating ways in which communities and their members can transform their beliefs, relationships and capabilities in ways that build their capacity to deal proactively with future hazard events [6,9,10,11]. Transformative learning facilitates people’s ability to make fundamental shifts in how they perceive themselves and their world, how they think, feel and act, how they relate to each other, and how they respond to novel environmental challenge and change [6,11,12,13]. This paper discusses two broad areas where transformative activities could be beneficial for DRR strategies.

The first relates to capacity building during disaster response. This section of the paper responds to calls from the UNISIDR [8] and international disaster recovery agencies to include capacity building in disaster response and recovery. That is, to develop future preparedness from intervention in recovery settings. The paper draws on analyses of several case studies to identify predictors of transformation and the emergence of enduring DRR capability. Developing a comprehensive approach to preparedness also calls for including transformative strategies in pre-event DRR (when most preparedness work occurs). 

The second major issue covered in the paper discusses why including a transformational component in DRR programs would be beneficial and how it can be developed. This discusses how DRR strategies can be developed in ways that include triggering citizen’s acceptance of a need for change and the inclusion of a mechanism that can ensure that can turn this recognition into new ways of knowing, thinking and acting that is shared by all stakeholders implicated in regional DRR processes. This draws on the Faultline Theory to introduce a potential catalyst for change during periods of hazard quiescence (when most preparedness work occurs) and then discusses how the scenario planning technique could be used to turn the potential for transformation created by Faultline principles into new ways of thinking and acting. The paper also discusses the how post-event and pre-event strategies can play complementary roles in comprehensive DRR. In the next section, a rationale for developing transformative processes in post- and pre-event settings is introduced. 

## 2. Developing Understanding of Transformative DRR

Internationally, calls for more attention to be directed to capacity building have been expressed via the build back better (BBB) and linking relief, rehabilitation and development (LRRD) concepts [8,14,15,16,17,18]. These calls emanate from, for example, recognition that prevailing low levels of preparedness in developed and developing countries alike are exacerbating the losses experienced when disaster strikes and extending recovery times. Furthermore, projected increases in the frequency and intensity of hazard activity will create circumstances in which humanitarian and government resourcing for response and recovery will be progressively stretched and eventually exceeded. Consequently, on humanistic and economic grounds, adding capacity building to response and recovery goals will have beneficial implications for communities and for response agencies [16]. 

A challenge in this context is identifying the social and community competencies that underpin people’s collective ability to collaborate in ways that generate new ways of thinking and acting [19]. One way of developing this capability is to examine how disaster-affected communities mobilize their social resources in circumstances where they cannot rely on external aid in order to facilitate their own recovery when faced with novel challenges. Developing this understanding can inform the community assessment and development strategies used to implement social BBB/LRRD strategies. 

To explore how communities and their members developed their “self-help” capability, this paper discusses examples of transformative outcomes that arose from people’s experience of disaster in Kagoshima (Japan), Simeulue (Indonesia), and Ho–Ping (Taiwan). These illustrative cases were selected because they represent areas where the authors have undertaken research into socio–cultural aspects of transformation and its influence on contemporary DRR practices [12,20,21,22,23]. The paper is not arguing that these are the only examples of transformative processes. Rather, the authors’ familiarity with them affords opportunities to illustrate how DRR capabilities emerged from community-based transformative processes and how these came to be sustained over time. 

The paper then contrasts these with cases the authors have worked on in Christchurch, New Zealand [1] and Tasmania, Australia [24]. The latter cases illustrate change via the emergence of new social processes which were not universally sustained beyond the recovery period for their respective disasters. 

Comparison of cases (Kagoshima, Simeulue and Ho–Ping) where emergent DRR processes were sustained with those in which emergent processes were short-lived (Christchurch, Tasmania) can offer insights into why some emergent DRR practices are sustained and some are not. Short-lived change does little or nothing to contribute to developing the capability to confront future events. However, knowledge of the factors that contribute to transformation in people’s beliefs and actions can inform the construction of strategies to develop sustained future capability. This is essential to developing community capability and reducing community reliance on external assistance [8]. The second area where DRR may benefit from including transformative activities concerns pre-event preparedness. Several factors drive the need to add a transformative dimension to DRR. The paper discusses how factors such as changes in hazard-scapes (e.g., from infrastructure development, increases in hydrological and meteorological hazards) and a consequent need for changes in socio–environmental relationships call for transformation in people’s thinking and behavior. 

It is also important to explore interdependencies between post-disaster transformation and the kinds of strategies that will be used in “pre-event” settings. For example, while BBB/LRRD strategies can lay a foundation for future capacity building, they cannot anticipate all future possibilities. Hence, it will be important to complement post-event initiatives with lessons from pre-event work to continue to develop community capability (e.g., from understanding the novel circumstances will face as a result of climate change). Conversely, pre-event strategies can benefit from lessons from communities faced with the reality of a need for change (e.g., developing more ecologically valid knowledge of change processes). Consequently, post-disaster and pre-event strategies can complement one another. 

While transformative pre-event strategies can profit from knowledge gained from the case study analyses presented in the discussion of BBB/LRRD capacity development (e.g., understanding social influences on transformation), pre-event programs face a challenge regarding the catalyst for change. In post-disaster settings, the catalyst for change is a disaster. This paper proposes using the generation of functional conflict as a substitute in pre-event settings. To do so, it draws on Faultline Theory [25]. 

Faultline Theory argues that change can be facilitated by mobilizing the diverse DRR beliefs and attitudes that prevail within neighborhoods of community groups (e.g., different beliefs about environmental protection, the need for preparedness, how much is enough, what form preparedness should take), but which are rarely the subject of social debate. Faultline Theory argues that bringing these diverse beliefs and views to the forefront of community discussion can stimulate conflict that can, if managed, act as a catalyst for transformation [11,25]. The paper then discusses how, if conflict is to generate transformational thinking and action, a mechanism for managing it create shared views and beliefs amongst all stakeholders is needed to create novel and functional DRR beliefs and goals. This paper draws on scenario planning (e.g., [26]) to present one such mechanism. Scenario planning is a developmental process designed to facilitate the development of coherent views amongst diverse stakeholders who are dealing with complex and uncertain circumstances. This makes it an appropriate tool to use in DRR contexts. 

Discussion commences with examples of emergent transformative process from Japan, Indonesia, Taiwan, New Zealand and Australia. The cases from Japan, Indonesian and Taiwan are discussed first. These are presented separately as they illustrate cases where emergent transformation occurred, and which was sustained over time (several years to decades). Then the New Zealand and Australian cases are introduced. The latter are discussed separately as they represent cases where emergent transformation occurred but was not sustained beyond the immediate recovery period. Separating the cases in this way afford a way to explore reasons why change is sustained in some cases but not others. The case studies illustrating sustained transformation are presented first. 

## 3. Transformation in Disaster Recovery: Developing Capacity and Capability

Kitagawa [21] presented an analysis of historical evidence from Kagoshima, Japan regarding how the Taisho eruption of Sakurajima volcano in 1914 became a catalyst for transformation in Kagoshima’s DRR policy and practice. She discussed how when reflecting on the impact the eruption had on the city, Kagoshima’s Mayor recognized how ignoring local knowledge of natural warning signs contributed to the death toll from the eruption. This led the Mayor to lead the development of new DRR principles [21]. The first change involved encouraging people to take responsibility for their own safety and to exercise this responsibility. This gave rise to the DRR principle of personal agency. A second principle derived from calls by a seismologist, Torahiko Terada, for people to become knowledgeable about volcanic processes and how to respond to volcanic hazards. The Mayor’s leadership facilitated a transformation; developing new DRR beliefs and practices built around these “agency” and “knowledge” concepts in ways that persist into present day DRR [21]. Another example of transformation emerged on the island of Simeulue in Indonesia. 

Kanamori et al. [20] discussed how, following islanders’ experience of a devastating tsunami in 1907, the Simeulue islanders developed a term, “smong,” that served as a tsunami-specific DRR process. Simeulue has an oral culture, with smong, as with other cultural processes, being sustained and passed from generation to generation by community elders through stories and song [23]. 

On Simeulue, the word “smong” describes tsunami event precursors and how to respond should they be detected [20,27]. The principle elements of smong [23] are: (1) Jika gempa kuat (If there is a strong earthquake), (2) Jika laut surut (If the sea recedes), (3) Lari ke gunung (Run to the mountains), and 3b Ngakk menunggu—lari saja! (Don’t wait—just RUN!). The smong story has become embodied in the socio–cultural fabric of Simeulue life in ways that influence how the islanders respond to tsunami [23]. 

The significance of smong as a DRR mechanism became apparent almost 100 years after the 1907 event when Simeulue was impacted by the 2004 Indian Ocean tsunami (Kanamori et al., 2010). Kanamori continues by discussing how, compared with neighboring islands and the Indonesian mainland, the death toll from the 2004 tsunami on Simeulue was very low. This low death toll was attributed to the smong narrative creating tangible actions; in 2004, most islanders recognized the tsunami threat and responded appropriately when it was detected [20,23]. The final example of sustained transformation presented originates in Taiwan. 

Paton, Jang and Liu [12] discuss how the residents of Ho–Ping township Taiwan experienced considerable damage from the 921 Earthquake of 1999. As a result, the township and its residents were cut off from the rest of the country for a prolonged period and had to develop new ways to organize their own response and recovery. Here, transformation took the form of developing a new socio–structural process to support rebuilding the community in the exceptional circumstances in which the villagers found themselves [12]. This emergent social structural process comprises four dimensions: community participation (e.g., organizing and participating in community activities), community consciousness (e.g., community development through engagement), trust in local and community organizations, and developing organizational networks (e.g., developing networks with NGOs and government agencies) [12]. 

It is important to appreciate that the processes evident in present-day Kagoshima, Simeulue and Ho-Ping did not always exist; they required transformation in how people thought about, related to and acted towards environmental hazards. In each case, social learning processes triggered by a disaster prompted the development of emergent community adaptive capacities that endured over time. In each case, the shifts in thinking and acting, and their consolidation into community life, occurred without external influence. These kinds of outcomes are what BBB/LRRD programs applied to community development intend to occur. Consequently, these cases can be analyzed with a view to identifying the social, community and governance processes that can be tentatively implicated as predictors of transformation in disaster recovery settings. This knowledge could inform ways to systematically and proactively develop transformative strategies to support building community capacity in BBB/LRRD programs. 

To pursue this goal, three basic types of information are needed from the analysis of the cases. The first concerns what motivates transformation. The second calls for identifying how recognition of this need translates into new ways of thinking and acting. This can be used by BBB/LRRD programs to assess communities and identify what social competencies and knowledge need to be developed to facilitate and support capacity building. The final issue is identifying the predictors of transformative processes being sustained over time as enduring adaptive capacities. The latter can inform how BBB/LRRD programs can be applied to the goal of increasing the likelihood of communities developing and maintaining the capabilities required to support their capacity to respond to disasters irrespective of when they occur. The importance of the last point derives from appreciating that emergent change can occur but need not be sustained over time. That this can occur is evident in the final two cases presented here from New Zealand and Australia. 

Emergent change was found in Christchurch, New Zealand following the 2011 earthquake [1] and in Tasmania, Australia following a wildfire disaster in 2013 [28]. However, neither in Christchurch nor Tasmania were these emergent capabilities sustained over time; they dissipated within a few months of the events occurring. Being able to compare cases where emergent DRR processes were sustained with those in which emergent processes were short-lived can offer additional insights not only into factors motivating the development of emergent capabilities, but also into those that determine whether post-event changes are converted into enduring social-structural processes. This is an important issue if UNISDR [8] calls for increasing capability building are to be realized. The discussion of these issues commences in the next section. 

While a disaster was the catalyst for transformation and shifts in beliefs in each case, it was not the disaster per se that stimulated change. Rather, it was the community characteristics, relationships and competencies required to ensure that the need for change is recognized and acted upon that emerged to respond to the circumstances that supported transformation. It is to a discussion of these factors that this paper now turns.

## 4. Community Leadership and Community Competencies

An understanding of the community characteristics, relationships and competencies that underpin emergent transformation can be developed by drawing on historical records and the findings of contemporary research into emergent transformation [1,12,20,21,23,28,29]. Collectively, these analyses identified how the emergence of novel ways to confront challenging circumstances is influenced by a mix of community leadership and community processes [1,21,24,29,30,31,32]. These community competencies are discussed in the next section, starting with the role community leadership plays in emergent transformation. 

Community-based leadership played a fundamental role in the emergent transformative outcomes observed in all the cases discussed [12,21,23,32]. The importance of leadership has been reinforced from studies of its absence. For example, following the 2011 Christchurch earthquake, interviews with members of well-established Residents’ Associations whose neighborhoods were affected discussed the failure of their Association to support its members’ recovery [1,31]. Association members attributed this to passive leadership on the part of the Resident Association leaders; the Association leaders chose to wait for government to advise what to do rather than taking the initiative to manage local response issues. In contrast, in functional neighborhood groups, leaders were willing and able to act in the atypical circumstances of the disaster and did so in ways that inspired people and empowered their creativity to support emergent, transformative outcomes [12,28,29,30,31,32,33]. Effective leaders were characterized as possessing: good local knowledge of people, place and local issues, needs and priorities; a strong sense of commitment to helping others within the community; knowledge of community functioning and how to empower community action; and they brought leadership and planning experience to their role [1,28,29,30,31,32,33,34]. 

It was evident in Kagoshima, Simeulue and Ho–Ping, that transformative outcomes became consolidated in adaptive capacities that endured over time [12,21,23]. While emergent group capability was evident in Christchurch and Tasmania, there was no evidence of the emergent social-structural capacities being sustained over the longer term once recovery had stabilized. This makes it pertinent to ask why transformative activities were consolidated into enduring adaptive capacities in some cases but not others. Answers to this question can inform understanding of how the development of enduring capabilities could be facilitated. This issue is examined from two perspectives. The first concerns the social processes implicated as predictors of transformation, and the second addresses leadership. 

The case analyses identified several community characteristics that underpinned the emergence of the functional relationships required for community groups to be able to support their own recovery. Prominent here were people’s willingness and ability to engage with others and maintain relationships (active community participation), being ready and able to share one’s stories and to listen to others’ stories, accommodating community diversity, being willing to engage with others to confront local issues (collective efficacy), having a sense of community within a neighborhood, and having a sense of attachment to place. These competencies influence people’s commitment to collaborate to develop local solutions to emergent problems [1,21,24,28,29,31,34]. Other predictors included a sense of group identity and trust in leaders and other community members. These factors combined to influence people’s commitment to planning and action [1,28,35]. The importance of identity was further reiterated in studies linking the development of social identity in recovery settings to empowering community action and effective post-disaster reconstruction [36,37]. 

In all the cases, transformation was supported by factors such as collaboration, cooperation and trust in confronting novel circumstances. There were thus no obvious differences in community characteristics that could be implicated in explaining differences in the conversion of emergent capabilities into enduring adaptive capacities. An alternative line of inquiry focuses on differences in the nature of the community-based leadership in the various locations. 

For example, in Kagoshima, Simeulue and Ho-Ping leaders were, respectively, the local mayor and community elders with responsibilities for managing community life before and after the disasters that occurred. Hence, the leaders in the Kagoshima, Simeulue and Ho–Ping cases were in positions in which they were responsible for developing and enacting the governance practices available to support transformative change. Their respective positions influenced their ability to ensure that the transformative outcomes that occurred were embedded in the socio–political context in the community settings in which DRR planning occurs and is put into practice. This may have contributed to the transformations occurring in the first place and to their being sustained over time. 

In contrast, in both the Christchurch and Tasmanian cases, the community leaders were emergent leaders. That is, they were not in official or representative positions where they could change or influence the development of the kinds of governance processes required to consolidate outcomes into enduring adaptive capacities. In fact, their roles often placed them in conflict with government leaders [30,31]. This limited the opportunities available to these community leaders to influence the development of the DRR governance structures required to ensure that socio–structural processes are sustained over time [38]. Thus, while change occurred, it lacked the leadership required to make it a transformative process. This proposition echoes Pelling’s [6] call for DRR governance structures and processes to be developed if transformative change is to be facilitated.

In all the case studies discussed above, an important competence was leader commitment to promoting inclusivity and ensuring that as many people as possible had roles to play in emergent recovery activities [1,24,28,31,32,39]. This highlights the importance of leaders acting to empower people in the transformative process. This also draws attention to a need to identify the community competencies required to ensure that leadership efforts invested in promoting inclusivity translate into meaningful collective transformation [1,32]. 

The importance of an inclusive and engaging approach to leadership was reinforced from finding that external leadership could act to hinder the development of functional capabilities in disaster-affected group [1,31]. A significant constraint on the development of functional capability derived from emergent relationships between community groups and the NGOs managing response activities during the response to the Christchurch earthquake. One group interviewed by Paton and colleagues commented on how they received external aid from an NGO immediately following the February 2011 earthquake [1]. This meant that they did not need to organize themselves to support their own recovery at that time. That is, top-down imposed leadership undermined the kind of natural recovery transformation observed in other groups. The significance of this disempowering action became evident following a significant aftershock in July 2011. Members of this group were faced for the first time with having to take responsibility for their own recovery [1,31]. They reported that, because of having to develop plans and actions for the first time, their recovery in July took several weeks. 

In contrast, groups who did not receive external assistance in February 2011, and who thus had to develop their own response capabilities, reported how the lessons learnt from their February experience allowed them to respond within days to the consequences they experienced in July 2011. Furthermore, they commented on how aftershocks provided opportunities for further refining and developing their emergent capabilities [1]. 

The discussion of factors that underpin emergent change (e.g., leadership, community participation, collective efficacy, sense of community, social identity) can inform the development of a framework for use in BBB/LRRD capacity building planning. The example in the previous paragraph from Christchurch regarding how NGO involvement can lead to diminished community capacity highlights the importance of including community empowerment as a principle in BBB/LRRD strategies. 

As outlined earlier, it is important to consider other issues that will affect the optimization of BBB/LRRD activities. For example, it is unlikely that a given event will encompass neither all possible future environmental challenges nor the consequences they will create for communities. For instance, DRR programs will need to continue to accommodate changes in community memberships and shifts in hazard-scape characteristics. While such issues are not considered in BBB/LRRD planning explicitly, optimizing the outcomes of BBB/LRRD initiatives will benefit by follow-up using preparedness strategies of the kind that will be used to facilitate pre-event preparedness. This point introduces the focus of the second area of transformation canvassed in the paper; pre-event transformation. 

The paper now turns to discussing what needs to be done to develop a proactive approach to transformative capacity building by including it in pre-event DRR strategies. In the Introduction to this paper, a need to rethink preparedness was raised. A rationale for this rethinking opens the discussion of pre-event strategies. The first issue here is why transformation may be beneficial. A second issue concerns identifying what could act as a catalyst for transformation. 

## 5. Transformation in Pre-Event DRR Settings

There exist several reasons for including transformative processes in DRR preparedness programs. All derive ultimately from the fact that levels of hazard preparedness are low [2,3,4,5,7]. These authors have identified that, for a substantial number of people, this relates less to the quality of contemporary risk communication and more to differences between risk communication authorities and citizens regarding how each think about their relationship with their environment and its potential hazards. These differences contribute to the challenges conventional risk communication faces when trying to facilitate preparedness. This paper next discusses three examples of factors that illustrate how citizens can think about hazards and preparedness in ways that differ substantially from their risk management professional counterparts. One concerns beliefs about socio–environmental–hazard relationships. Another focuses on increasing people’s acceptance of novel hazards. The third centers on the fact that while some people decide to prepare, others decide not to prepare for hazard events. 

### 5.1. Socio–Environmental Relationships

Fundamentally, disasters are events of human origin. The nature and location of societal development often reflects how geological, hydrological and other natural processes create the resources, amenities and facilities (e.g., fertile soils, natural harbors, navigable rivers used for transport and trade, forests that supply wood products, water supplies, coastal and mountain scenery etc.) that support societal vitality. However, what people do to secure the benefits from these environmental resources and amenities (e.g., where and how they build cities, develop economies through environmental resource exploitation, develop on flood plains, deforestation etc.) make significant contributions to the natural hazard risks posed to societies and their citizens (Paton, 2017). Acknowledgement of this relationship underpins recognition of a need for DRR policies, plans, and applications to accommodate social-environmental–hazard interdependencies [40,41]. A transformative DRR issue arises here because societies (and their members) differ regarding how they construe social-environmental–hazard interdependencies. In some cases, they are construed in ways that create a disconnect between these social, environment and hazard elements. This is evident when the prevailing western view of this relationship is considered. 

Western conceptualizations of socio–environmental–hazard relationships have favored an anthropocentric perspective; people see themselves as independent from their natural environment and see is as a resource for economic gain and exploitation. Regarding its implications for DRR, anthropocentric approaches focus attention on control over the environment, with citizens transferring responsibility for control from themselves to societal institutions and agencies [42,43,44]. The control strategies adopted by these agencies typically rely more on technical (e.g., warnings) and engineered (e.g., levees) mitigation than on including activities to increase people’s sense of responsibility for their own risk management [5,10,45]. 

People’s transfer of responsibility for DRR from themselves to civic and scientific agencies lessens the likelihood of their supporting UNISDR calls for shared responsibility approaches to DRR [8,46]. In DRR settings, this is especially problematic when people overestimate the effectiveness of technical and engineering mitigation and underestimate the complementary need for their playing a role in their DRR. For example, structural earthquake mitigation in apartment buildings increases the resilience of the building, but if people do not secure internal furnishings and fittings in their individual apartments, they risk being killed or injured when these are dislodged by ground shaking. Government agencies can ensure that building codes are in place, but residents need to take responsibility for securing the contents of their apartment. This makes it important to facilitate people’s appreciation of the need for shared responsibility. One way of pursuing this goal is through transforming how people conceptualize their relationship with their environment and its potential hazards. That such a transformation is possible was evident from events in Kagoshima (see above). 

In Kagoshima, following the Taisho eruption, recognition of how over-reliance on scientific warning sources contributed to the losses experienced prompted a need for greater emphasis on local knowledge, with this being translated into DRR through the principles of *agency* and *knowledge* [21]. Kitagawa continued by discussing how the introduction of these principles, particularly that concerning environmental knowledge, prompted changes in how people conceptualized their socio–ecological relationships. 

In Kagoshima, calls for people to become more knowledgeable about their environment and its hazards underpinned the development of the construct of kyozon [21]. This construct describes a mechanism that facilitates socio–ecological co-existence with the implications of the frequently erupting Sakurajima volcano. The development of this construct enabled reconciling the socio–environmental costs and benefits arising from living adjacent to a highly active volcano [21]. This opens the discussion to considering the potential for changing socio–ecological beliefs and actions. This is reinforced by comparison between western views and the more ecocentric perspectives adopted by Indigenous peoples and those in many Asian countries [42]. Other emergent co-existence strategies are evident elsewhere in Japan. 

In Japan, the Machizukuri (community led place-making with care) construct (e.g., [47]) promotes environmental co-existence and adaptive practices through functional partnerships between civic agencies, urban planners and citizens (i.e., it facilitates shared responsibility). This empowers people to reconcile social, environmental and DRR goals [48]. Machizukuri is also influential in reconciling the needs and goals of diverse stakeholders in ways that culminate in their developing novel, inclusive disaster recovery initiatives [22]. The Machizukuri process operates in a comparable way to the scenario planning technique introduced below. This offers support for the potential of scenario planning to play a role in DRR (e.g., facilitating functional socio–ecological relationships). Other examples of ecocentric beliefs with DRR implications are evident in Indonesia and China. 

In Indonesia, the socio–cultural construct of Palemahan identifies the important role positive relationships between people and their natural world play in DRR [49]. Paton and Sagala discuss how this relationship manifests itself in several socio–cultural practices that encompass environmental co-existence and shared responsibility, including the Subak traditional community-based water management process, the traditional agrosystem in Baduy, and the influence of Musalaki (cultural leadership) on people’s response to environmental challenge and change. 

In Chinese culture, beliefs regarding the maintenance of harmonious (co-existence) relationships between people and nature is a fundamental tenet of Confucian ethics. This ethic can influence people’s quality of life [50] and their engagement in developing strategies to facilitate resilience to environmental hazards [51]. Similar findings have emerged in research with Indigenous peoples [42]. 

These examples illustrate how an ecocentric conceptualization of socio–environmental–hazard interdependencies can mobilize collective stakeholder DRR actions that reconcile environmental costs (from hazardous events) and benefits (taking advantage of environmental opportunities and amenities). Transforming western anthropocentrism into a more ecocentric conceptualization of socio–environmental–hazard relationships could thus contribute to developing more effective and sustainable DRR outcomes [8,11,40,41,42]. Transforming socio–ecological relationships in ways comparable to those outlined above will become increasingly important as climate change increases the distribution, intensity and frequency of hazards from storm, hurricane (cyclone/typhoon) and wildfire hazards. Thus, DRR processes will benefit from people having a better understanding of how their relationship with their environment affects their risk [41]. This improved understanding, in turn, can lay a foundation for more effective socio-environmental preparedness [10]. More comprehensive socio-environmental understanding can facilitate citizen appreciation of the need to prepare to confront hazard activity from sources they have not previously had to deal with. This last point introduces the next sub-section. 

### 5.2. Transformation and Dealing with Change in Local Hazard-Scapes 

The authors have been involved in several projects investigating preparedness in communities where the hazards people are being encouraged to prepare for are very infrequent. For example, in Australia, this includes encouraging community tsunami and earthquake risk acceptance in states (e.g., Tasmania, New South Wales) where these hazards are rare but capable of creating highly significant impacts on communities with little or no warning [52,53]. A significant issue in this context is the fact that the low return periods of earthquake and tsunami hazards in Australia means that the risk posed by these hazards is either denied or under-appreciated. 

Research in the locations in the previous paragraph identified how the construct of risk rejection (a disbelief in the presence of the source of the hazard) underpinned people’s DRR thinking about tsunami and earthquake hazards [53,54]. This acts as a significant impediment to preparedness that cannot be overcome using traditional risk communication. Transformative change is required to provide a more neutral starting point for DRR for these events [55]. 

A need to facilitate people’s appreciation of the need to prepare for novel hazards is occurring in other locations. For example, a need transformative processes to facilitate people’s acceptance of the emergent risk (a consequence of climate change) posed by wildfire hazards has been recognized in Taiwan [56]. Changes in hazard-scapes will occur in many locations as a result of climate change, increasing the international benefits that could accrue from including a transformative perspective in DRR planning. Another example of the benefit of including a transformative component in DRR derives from finding that some citizens acknowledge their risk, they nonetheless decide not to prepare for the hazards from which the risk is sourced. 

### 5.3. Hazard Beliefs and Cognitions

Most of the practical work on preparedness assumes that people are predisposed to prepare and that identifying and applying strategies derived from its (theoretical) antecedents will automatically increase preparedness over time. However, as several authors discuss, this assumption can only be partially justified [2,3,4,5,7,43,57,58]. One reason for this is that some people decide not to prepare [2,43,59]. Indeed, the numbers of people deciding not to prepare may be substantial. 

For example, a study of volcanic preparedness in Auckland, New Zealand [60] discussed how 94% of a sample of 400 people participating in a study evaluating the effectiveness of a volcanic hazard preparedness program did not prepare. This was not due to people’s ignorance of their volcanic risk; some 92% of those surveyed acknowledged their potential exposure to volcanic hazard events. Furthermore, some 63% (i.e., 237 out of the 400 people surveyed) of those who had not prepared stated that they had no intention of preparing (data were obtained following a volcanic hazard public education program advising residents of Auckland of their risk and their need to prepare) in the future. This study identified how the cognitive biases of unrealistic optimism and risk compensation [40,60] underpinned people forming intentions to do nothing (see below). 

If these statistics are extrapolated to the population at large, the number of non-preparers could run into hundreds of thousands of Aucklanders. This increases the risk faced by a substantial number of people and it will, when an eruption occurs, place additional, but potentially avoidable, demands on recovery resources and agencies. This is not the only data from governmental sources identifying non preparedness. Some 12 months following the 2011 Christchurch earthquake, Statistics New Zealand [61] identified that 75% of those surveyed in areas with continued exposure to aftershocks had not undertaken any kind of preparation. 

The hazard literature includes several factors that assist understanding why some people decide not to prepare. These include dispositional factors and cognitive biases such as denial, avoidance, hazard-related anxiety/fear, unrealistic optimism, and risk compensation [40,55,62,63]. Other studies identified how people’s interpretation of hazards can interact with psychological, social, institutional, and cultural processes to attenuate people’s (individual and collective) interpretation of risk, with this culminating in their deciding not to prepare [44,63,64]. The influence of one or more of these factors and processes can lead to “not preparing” becoming entrenched in people’s hazard-related beliefs and result in those who “prepare” and those who decide “not to prepare” differing considerably in how they think about preparing [62,63,64,65]. 

In the McIvor et al. [62] and Paton et al. [65] studies, those who prepared believed that preparing could enhance their safety, had a strong attachment to where they lived, a high level of involvement in their neighborhood and community and a strong sense of social responsibility to others in their community, and they did not believe that insurance would compensate for hazard-related losses. In contrast, non-preparers did not believe that preparedness could enhance their safety, felt no or little sense of attachment to where they lived, had little sense of social responsibility to others, and believed that insurance would cover any losses incurred. 

The evident differences between preparers and non-preparers means that traditional preparedness strategies are more likely to fail for those people (and, as the above data suggest, this could be a substantial number) whose underlying beliefs do not support preparedness as a DRR option. The above discussion suggests that “preparing” and “not preparing” outcomes do not lie at opposite ends of a continuum. Rather, they are separate processes and need to be managed as such in DRR strategies [43,64]. Consequently, it would be beneficial to develop DRR strategies geared towards transforming the beliefs of those whose current default option is to “not prepare.” Taken together, the above examples provide a rationale for why adding a transformative component to DRR could be beneficial (e.g., increased shared responsibility, better understanding of hazard–environment relationships, more informed foundation for hazard preparedness). This process can capitalize on some of lessons learned from the analysis of transformation in post-disaster settings. 

The analyses of the cases in Kagoshima, Simeulue, Ho–Ping, Christchurch and Tasmania [1,12,21,23,28] provided evidence that transformation was a social process. That is, the relationship between a disaster (the catalyst) and new, transformative ways of thinking and acting was mediated by social factors such as community leadership, active community participation, collective efficacy, sense of community, social identity and trust (see above). Parallels between the mediating process identified in disaster-related transformation and those in theories of pre-event preparedness are evident [11,43,66,67,68]. This provides tentative support for building on preparedness theories to develop models of transformative change. 

However, it should be noted that factors such as a catalyst for change and key drivers of transformation such as community leadership need to be included and given a more pivotal role in a model of transformative change. The role of leadership will be discussed later. First, if a transformative element is to be introduced into DRR, what is needed is a strategic process that challenges pre-existing beliefs and does so in ways that motivates change in most or all stakeholders. 

Studies of social transformation identify the importance of some “provocation” [69]. This represents a trigger or catalyst that functions to call the prevailing status quo into question in a way that is seen as challenging by all or most of those affected and that draws attention to a need for new ways of thinking and acting [69]. In the cases discussed above, the provocation or catalyst for transformation was a disaster. If practical transformative strategies are to be introduced into DRR settings it requires the development of some kind of “provocation” capable of motivating not only new ways of thinking and acting, but also ensuring that these are shared and acted on by stakeholder groups characterized by considerable diversity. 

## 6. Mobilizing Transformative DRR

If transformative change is to occur in pre-event DRR settings, a catalyst or provocation capable of motivating new ways of collective thinking and acting is required [69,70,71]. The challenge for DRR is how to create this. This paper discusses one approach to doing so. This involves first encouraging conflict by mobilizing the diverse knowledges, beliefs, goals and relationships that exist within neighborhood and community groups to increase the range of issues and needs brought to public awareness. Then the strategy must channel the conflict that arises from this mobilization in ways that create a framework for transformation that encompasses the needs and goals of, as far as possible, all stakeholders. First, the foundation for mobilizing shifts in this and action is illustrated using Faultline Theory [25,71].

### 6.1. Faultline Theory: Providing a Catalyst for Change

Despite its name, Faultline Theory has nothing to do with seismic processes. It describes how fundamental differences in community members beliefs and attitudes (e.g., about environmental protection, the efficacy of hazard preparedness etc.) can exist within groups (e.g., families, neighbors, community members, work colleagues etc.) but have no influence on the quality of relationships between group members under normal circumstances. However, if an external condition elevates the relative salience of the different beliefs that exist within a group, this triggers intra-group conflict [25,70,71,72]. Belief differences thus represent “faultlines;” attitudinal phenomena that are generally inactive, but which can be activated if an external stressor acts upon them.

For example, Paton and Buergelt [70] discussed how a neighborhood at-risk from wildfire hazards comprised people with different underlying beliefs. Some held relatively strong environmental beliefs. Others placed relatively higher value on household safety. Under normal conditions, these diverse beliefs were benign; the different beliefs had no bearing on the quality of relationships between neighbors. However, government calls for property vegetation clearing changed this. This external condition acted as a catalyst that brought different beliefs to the surface of everyday social interaction and resulted in these beliefs acting to adversely affect neighbor relationships. For instance, those valuing environmental protection (opposed to vegetation clearing) over safety found themselves in (often significant) conflict with those who valued household safety (supporting vegetation clearing) over environmental protection. Hence a faultline (the emergence of very different views about environmental versus safety issues in a public forum) was activated, generated dysfunctional conflict, and reduced people’s commitment to prepare. 

However, if this conflict is actively managed within a DRR program, this kind of attitude mobilization can act as a catalyst to facilitate transformation [71]. For example, deliberately mobilizing the respective beliefs of those valuing environmental protection and those valuing household safety in a public debate could create a context in which people become aware of a need to accommodate both in new conceptualizations of comprehensive community-based DRR activities. For this to occur in practice, a method for managing functional conflict and constrictively facilitating reconciliation of diverse needs is required. This calls for the inclusion in DRR strategies of a mechanism that affords people opportunities to appreciate both how the beliefs and goals of all stakeholders are important and why they need to be integrated to generate new ways of thinking and acting that are beneficial for all stakeholders in DRR. That is, the process must generate a context in which residents can collaborate to co-create outcomes that are relevant for all stakeholders [73,74]. The earlier discussion of Machizukuri introduced how social processes are capable of successfully eliciting the views and goals of diverse stakeholders and reconciling them in ways that create novel and inclusive environmental beliefs, goals and actions. In the next section, the paper introduces scenario planning as a way of achieving comparable outcomes. 

### 6.2. Functional Conflict Resolution with Multiple Stakeholders: Scenario Planning

Scenario planning describes a method for facilitating planning in contexts where multiple stakeholders collaborate to confront external circumstances that are complex, dynamic and involve considerable uncertainty [26,70,75,76]. Scenario planning is valuable in circumstances in which stakeholder’s respective expertise, beliefs, needs and goals must be reconciled to create shared understanding of future DRR issues. Scenario planning thus creates a super-ordinate context for developing new DRR beliefs and practices. The process is founded on environmental analysis (e.g., around the hazards in a specific location), with this providing a foundation for collective stakeholders’ engagement in DRR planning. This increases its utility as a mechanism that can contribute to the goal of transforming socio–ecological beliefs. These characteristics make it an appropriate tool to use to provide diverse stakeholders with opportunities to engage to use their collective differences in hazard beliefs and goals as a context and foundation for co-creating transformative approaches to DRR. 

Stakeholders are first invited (through a facilitated process) to be creative in anticipating the hazard consequences that could affect them and where they live over the coming decades. The perspectives each stakeholder group generates are then shared amongst all stakeholders to increase the breadth and depth of collective stakeholder needs, goals, circumstances. These represent the scenarios available to stimulate people’s recognition of the need for new and inclusive ways of thinking and acting. 

Well-constructed scenarios challenge “tunnel vision” by instilling a deeper appreciation for the diverse factors that influence collective future risk. Scenarios go beyond the objective (the typical focus in mainstream risk communication) to elicit subjective interpretations that seek to capture the novel and diverse possibilities that drive from eliciting and sharing diverse stakeholder perspectives. These create the raw material from which novel and shared understandings of how to conceptualize and manage natural hazard risk are forged. 

By capturing the diverse views, beliefs, ideas, suggestions, experiences of all stakeholders, and making them available to all, scenarios create a planning context that encompass elements that extend beyond those of any one stakeholder group. The collective exposure to diverse beliefs, knowledges, relationships challenges the prevailing mindsets of all stakeholders. Scenario planning can thus act as a catalyst for transformative thinking and acting (i.e., it is a tool for collective learning and reframing perceptions) in ways that assist integrative planning for novel and uncertain future circumstances [26,70,75,76]. 

The effectiveness of scenario planning activities can be increased by providing community leaders with training in the kinds of transdisciplinary skills that can support collaborative and cooperative planning [32,74]. By organizing this information into narratives that are shared by stakeholder and that facilitate the collective development of a super-ordinate community identity [71], the scenario planning process identifies how stakeholders can draw on their respective contributions to work collaboratively to develop local DRR plans and strategies. 

Collaboration is important. It is fundamental to the task of constructing new, shared mental models and supporting shared responsibility for action. It also enables participation in decision processes, increases risk acceptance and collective ownership of problems and solutions, and facilitates identification of acceptable risk management options and their implementation in ways that contribute to obtaining outcomes that are meaningful for community members [71]. At a practical level, several approaches can be applied to facilitating collaboration. Examples include brainstorming, the Delphi method, extended contact, multiple classification training, and cooperative learning. 

Techniques such as brainstorming can be used if relationships are neutral or where levels of conflict are minimal. This allows the process to operate with people participating face-to-face. An alternative approach that can be used when moderate levels of conflict are evident is extended contact [77]. 

Extended contact enhances the quality of interpersonal relationships prior to people having direct contact by providing stories of interaction with others in similar circumstances (e.g., how other people reconciled environmental beliefs and wildfire mitigation). An example of the application of this approach to DRR involves challenging negative outcome expectancy (NOE) beliefs and transforming them to create a neural starting point for hazard preparedness strategies. 

Some people who do not prepare do so because they form “negative outcome expectancies” [55]. This way of thinking arises when people conflate their inability to influence the causes of hazard events (e.g., they cannot affect the causes of earthquakes or other natural processes) with an inability to act to reduce the consequences they could experience if earthquakes occur [78]. 

One way such beliefs can be transformed is by presenting information about preparedness and its effectiveness sourced not from experts, but from members of other communities that have prepared. Stories from sources that people more readily identify with (i.e., people like them) can act as a catalyst for their developing more neutral views on preparedness. This neutral position provides a foundation for increasing people’s receptiveness to preparing [55].

These techniques build a framework in which different views can be reconciled to create a super-ordinate approach to DRR planning. However, if the process elicits relationships characterized by highly dysfunctional conflict a better starting point is the Delphi technique [70]. The Delphi approach involves stakeholders providing their respective inputs initially via a facilitator (using the process outlined above). 

The facilitator then shares all perspectives amongst all stakeholders. This exposes member of the constituent stakeholder groups to different views, needs and knowledge of what they future may hold. By providing collective exposure to diverse and novel possibilities, the process challenges the mindsets of all stakeholder groups. The process continues through similar cycles, with the facilitator role helping to progressively develop shared understanding of the value and importance of collective input to defining future scenarios. Once a level of shared understanding has been reached, stakeholder groups can be brought together to complete the scenario planning process. 

Brainstorming, extended contact and the Delphi method expose stakeholders are techniques designed to expose stakeholders to diverse perspectives, allow them to evaluate ideas in general, and enable their developing consensus around new ways of thinking and acting. The collaboration engendered by these approaches can be further developed using methods such as multiple classification training [77]. 

Multiple classification training encourages people to shift from thinking about others along a single dimension to construing them in multi-dimensional ways (e.g., not only as an environmentalist but also as a local resident, a parent, a gardener, a community volunteer etc.). Cameron and Turner [77] discuss how this process reduces barriers between people, increases opportunities for their developing a shared identity, and creates a foundation for people to appreciate how diverse perspectives can facilitate the emergence of outcomes that are both novel and mutually beneficial to all stakeholders. 

Another approach involves cooperative learning [46,77,79]. Cooperative learning facilitates collaborative activities in which participants are given different segments or parts of the DRR puzzle and then working collectively to develop comprehensive community-based DRR process. For example, some stakeholders would work on the community relationship aspects of preparedness, others would focus on community–agency preparedness [46]. These components are then integrated to enable the development of comprehensive DRR plans [46]. If the ideas outlined here are to be systematically examined, the next step is to develop a model that can be used to formulate the hypotheses and research questions that inform future research into transformative DRR. A tentative model is discussed in the next section. 

## 7. Modelling Transformative and Adaptive Processes in Pre-Event Settings

**Figure 1:**
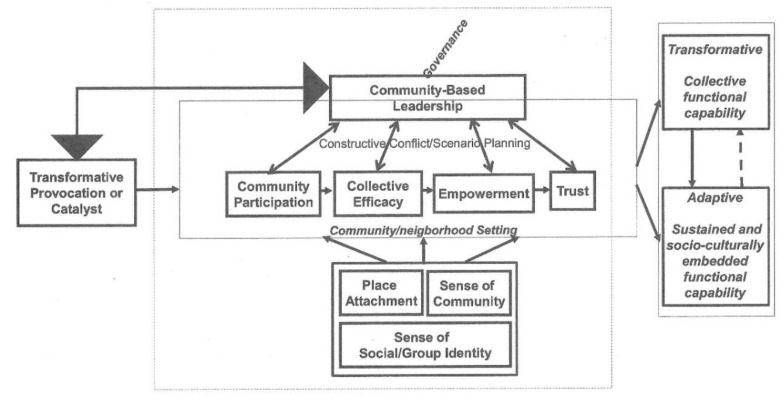
A model integrating constructs implicated in transformative outcomes and hazard preparedness.

Parallels between the socio–structural processes identified in the case study analyses (see above) and comparable constructs in preparedness theories such as the Community Engagement Theory (CET) [46,78] offer scope for developing a model of transformation in pre-event settings by adapting existing theory. A preliminary model derived from this integrative approach is depicted in Figure 1. 

Figure 1 describes the end-points (potential dependent variables in future research) comprising separate, but related outcomes; creating transformative outcomes and their consolidation into sustained adaptive capacities. The antecedents of these outcomes comprise the core components of the CET and tentatively add variables identified in the cases, but which were not in the CET. 

In Figure 1, the central part of the figure describes the core elements from the CET [78]. These reflect those implicated in transformation in the case analyses (see above). The fact that these elements emerged in communities actually facing a need to change adds to the value of building on the CET to develop a preliminary model of pre-event transformation. However, the case analyses identified factors that have not played prominent roles in preparedness theory and practice. These include a need for a transformative catalyst, community-based leadership, place attachment, sense of community and social identity. These are included in the tentative model depicted in Figure 1 by the relationship between leadership and community processes being mediated by “constructive conflict/scenario planning.” 

In the model, a pivotal role is played by the addition of community-based leadership. This is depicted to reflect it maintaining a reciprocal relationship with the provocation or catalyst for transformation and all community competences required to ensure that leader efforts to facilitate inclusivity are more likely to be realized in the process of facilitating transformative outcomes and their conversion into enduring adaptive capacities. 

To accommodate the possibility that leader effectiveness in converting transformative outcomes into more enduring adaptive capacities was linked to their influence of future governance (see Section 4), a role for governance was added to Figure 1 as an overarching process linked to community-based leadership. The inclusion of governance here reflects its being implicated as a factor in translating transformative change into enduring adaptive capacities in the Japanese, Indonesian and Taiwanese cases. The need for this addition to the model is reinforced by calls for community leaders becoming stakeholders in the DRR governance processes developed by local and national governments [32,38].

The solid line from transformative to adaptive outcomes depicts the former being a precursor of the latter. The dashed arrow from adaptive to transformative outcomes depicts how adaptive capacities will progressively contribute to the community capacities that support transformation and the emergence of new DRR beliefs and actions. Figure 1 thus describes a preliminary model that could be used to formulate research questions about transformative outcomes and their consolidation into enduring adaptive capacities. 

## 8. Conclusions

Low levels of hazard preparedness in areas increasingly susceptible to experiencing disaster has been identified as contributing to the scale of losses experienced when disaster strikes and with extending recovery periods [8]. This paper discussed how traditional approaches to risk communication are ill-suited to managing constraints to preparedness arising from, for example, issues with people’s socio-environmental relationships, their tendency to transfer responsibility for risk management from themselves to the authorities, and belief systems that support “not preparing” as the default response to DRR strategies. Because fundamental shifts in people’s thinking and acting are required, it was proposed that enhancing levels of preparedness calls for rethinking how preparedness is conceptualized and facilitated through transformative learning. This paper discussed two basic approaches to situating transformation in DRR. One approach involves including transformative strategies in post-event intervention. This entails complementing BBB and LRRD activities with social capacity building programs within disaster recovery and rebuilding activities. A second approach involves including transformative elements in pre-event preparedness DRR activities. 

The paper drew on work identifying where transformation occurred following disaster to identify predictors of transformation. These case analyses identified how a disaster could create a catalyst for transformation, with the relationship between this catalyst and new ways of thinking and acting being mediated by several social competencies (leadership, active community participation, collective efficacy, sense of community, social identity and place attachment). 

The paper discussed how while transformative outcomes can occur following disaster, this outcome is not inevitable; some groups fail to develop emergent capability. It was also evident that some groups develop this capability and convert into enduring adaptive capacities, while others develop emergent capability, but do not maintain it over time. Future work on understanding both how emergent, transformative outcomes arise and how they become sustained as adaptive capacities thus becomes important. 

One explanation advanced to account for this difference derived from differences between cases regarding the roles that community leaders play in DRR governance and hence became better able to influence the development of the governance processes required to sustain emergent socio–structural changes. Future research should thus pursue Pelling’s [6] call for DRR governance structures and processes to be developed if transformative change is to be facilitated. 

Including processes to facilitate both transformation and consolidating (and applying) the outcomes into adaptive capacities via preparedness strategies could have benefits for DRR planning and intervention. In the more dynamic hazardous futures that people the world over face a need to include both transformative and consolidating strategies will increase. They will be needed to, for example, accommodate changes in local hazard-scapes (e.g., in response to climate change consequences, seal-level rise, environmental pollution). Transformation and consolidation strategies will also be required to accommodate social (e.g., population and infrastructure development, migration, and changes in community memberships, goals, needs over time) inputs into the social-environmental–hazard relationship. The factors implicated as precursors of transformation were summarized in the development of a tentative model. 

## References

[1] Paton D., Johnston D., Mamula-Seadon L., Kenney C.M., Kapucu N., Liou K.T. (2014). Recovery and Development: Perspectives from New Zealand and Australia. Disaster & Development: Examining Global Issues and Cases.

[2] Harries T. (2008). Feeling secure or being secure? Why it can seem better not to protect yourself against a natural hazard. Health Risk Soc..

[3] Johnson B.B., Nakayachi K. (2017). Examining associations between citizens’ beliefs and attitudes about uncertainty and their earthquake risk judgments, preparedness intentions, and mitigation policy support in Japan and the United States. Int. J. Disaster Risk Reduct..

[4] Levac J., Toal-Sullivan D., OSullivan T.L. (2012). Household emergency preparedness: A literature review. J. Community Health.

[5] Lindell M.K., Arlikatti S., Prater C.S. (2012). Why do people do what they do to protect against earthquake risk: Perception of hazard adjustment attributes. Risk Anal..

[6] Pelling M. (2011). Adaptation to Climate Change: From Resilience to Transformation.

[7] Solberg C., Rossetto T., Joffe H. (2010). The social psychology of seismic hazard adjustment: Re-evaluating the international literature. Nat. Hazards Earth Syst. Sci..

[8] UNISDR (2015). Sendai Framework for Disaster Risk Reduction 2015-230. http://www.unisdr.org/files/43291_sendaiframeworkfordrren.pdf.

[9] Adger W.N., Dessai S., Goulden M., Hulme M., Lorenzoni I., Nelson D.R., Naess L.A., Wolf J., Wreford A. (2009). Are there social limits to adaptation to climate change?. Clim. Chang..

[10] Paton D., Paton D., Johnston D.M. (2017). Co-existing with Natural Hazards and their Consequences. Disaster Resilience: An Integrated Approach.

[11] Paton D., Buergelt P.T., Daniels J.A. (2017). Facilitating Social-Environmental Adaptation to Environmental Hazards: Towards a Universal Theory. Advances in Environmental Research.

[12] Paton D., Jang L.J., Liu L.W., James H., Paton D. (2016). Long Term Community Recovery: Lessons from earthquake and typhoon experiences in Taiwan. The Consequences of Asian Disasters: Demographic, Planning and Policy Implications.

[13] Pelling M., O’Brien K., Matyas D. (2015). Adaptation and transformation. Clim. Chang..

[14] Buchanan-Smith M., Fabbri P. (2005). Links between Relief, Rehabilitation and Development in the Tsunami Response: A Review of the Debate.

[15] Christoplos I. (2006). Links between Relief, Rehabilitation and Development in the Tsunami Response: A Synthesis of Initial Findings.

[16] (2013). International Recovery Forum Recommendations for Recovery and Reconstruction in Post-2015 Global Framework for DRR.

[17] Kapucu N., Liou K.T. (2014). Disaster and Development: Examining Global Issues and Cases.

[18] Mosel I., Levine S. (2014). Remaking the Case for Linking Relief, Rehabilitation and Development.

[19] Paton D., James H., James H., Paton D. (2016). Identifying New Directions in Post-Disaster Livelihood, Resilience and Sustainability in Asia. The Consequences of Asian Disasters: Demographic, Planning and Policy implications.

[20] Kanamori H., Rivera L., Lee W.H.K. (2010). Historical seismograms for unravelling a mysterious earthquake: The 1907 Sumatra Earthquake. Geophys. J. Int..

[21] Kitagawa K. (2015). Living with an active volcano: Informal and community learning for preparedness in south of Japan. Adv. Volcanol..

[22] Paton D., Jang L.J., Kitagawa K., Mamula-Seadon L., Sun Y., Paton D., Johnston D.M. (2017). Coping with and Adapting to Natural Hazard Consequences: Cross cultural perspectives. Disaster Resilience: An Integrated Approach.

[23] Sutton S., Buergelt P.T., Paton D., Sagala S., Paton D., Sagala S. (2018). Cultural Drivers of Disaster Risk Reduction Behaviour: The case of Pulau Simeulue. Disaster Risk Reduction in Indonesia.

[24] Paton D., Irons M. (2016). Communication, Sense of Community and Disaster Recovery: A Facebook case study. Disaster Communications. Front. Commun..

[25] Lau D., Murnighan J.K. (2005). Interactions within groups and subgroups: The dynamic effects of demographic faultlines. Acad. Manag. J..

[26] Ogilvy J., Nonaka I., Konno N. (2014). Toward Narrative Strategy. World Futures.

[27] Centre for Disaster Preparedness (2011). Indigenous Knowledge for Disaster Risk Reduction: Good Practices and Lessons Learned from Experiences in the Asia-Pacific Region. https://issuu.com/cdpfoundation/docs/unisdr_indigenous_knowledge-drr/30.

[28] Paton D., Jang L.J., Irons M., Brown D. (2015). Building Capacity to Adapt to the Consequences of Disaster: Linking Disaster Recovery and Disaster Risk Reduction. Capacity Building: Planning, Programs and Prospects.

[29] Paton D., Jang L.J., D’Amico S. (2016). Earthquake Readiness and Recovery: An Asia-Pacific perspective. Earthquakes and their Impact on Societies.

[30] Irons M., Paton D., Paton D., Johnston D.M. (2017). Social Media and Emergent Groups: The impact of high functionality on community resilience. Disaster Resilience: An Integrated Approach.

[31] Mamula-Seadon L., Selway K., Paton D. (2012). Exploring Resilience: Learning from Christchurch communities. Tephra.

[32] McAllan C., McAllan V., McEntee K., Gale W., Taylor D., Wood J., Thompson T., Elder J., Mutsaers K., Leeson W. (2011). Lessons Learned by Community Recovery Committees of the 2009 Victorian Bushfires. Cube Management Solutions.

[33] Behling O., McFillen J.M. (1996). A syncretical model of charismatic/transformational leadership. Group Organ. Manag..

[34] Jang L., LaMendola W., Paton D., Johnston D. (2006). The Hakka spirit as a predictor of resilience. Disaster Resilience: An Integrated Approach.

[35] Winstanely A., Cronin K., Daly M. (2011). Supporting Communication Around the Canterbury Earthquakes and Other Risks.

[36] Drury J., Jetten J., Haslam C., Alexander S.H. (2012). Collective resilience in mass emergencies and disasters: A social identity model. The Social Cure: Identity, Health and Well-Being.

[37] Farida A. (2014). Reconstructing social identity for sustainable future of Lumpur Lapindo victims. Procedia Environ. Sci..

[38] Mamula-Seadon L., Paton D., Sheng-Her R., Jang L.J. (2018). Building Community Resilience through Empowerment: Place-making in Different Cultural Contexts. Community-Based Disaster Risk Reduction and Recovery: Integrating Community Development and Risk Management.

[39] McLennan J., Elliot G. (2012). Community Bushfire Safety Issues: Findings from interviews with residents affected by the 2009 Victorian bushfires. Bushfire Cooperative Research Centre Fire Note.

[40] Buergelt P.T., Paton D. (2014). An ecological risk management and capacity building model. Hum. Ecol..

[41] Twigg J. (2015). Disaster Risk Reduction.

[42] Buergelt P.T., Paton D., Sithole B., Sangha K., Prasadarao P.S.D.V., Campion L., Campion J., Paton D., Johnston D.M. (2017). Living in Harmony with our Environment: A paradigm shift. Disaster Resilience: An Integrated Approach.

[43] Paton D., Smith L., Johnston D. (2005). When good intentions turn bad: Promoting natural hazard preparedness. Aust. J. Emerg. Manag..

[44] Paton D., Buergelt P.T., Campbell A., Daniels J.A. (2015). Learning to Co-Exist with Environmental Hazards: Community and societal perspectives and strategies. Advances in Environmental Research.

[45] Paton D. (2000). Emergency Planning: Integrating community development, community resilience and hazard mitigation. J. Am. Soc. Prof. Emerg. Manag..

[46] Paton D., Kerstholt J., Skinner I., Paton D., Johnston D.M. (2017). Hazard Readiness and Resilience. Disaster Resilience: An Integrated Approach.

[47] Kobayashi I. (2007). Machizukuri (Community Development) for recovery whose leading role citizens play. J. Disaster Res..

[48] Mavrodieva A.V., Daramita R.I.F., Arsono A.Y., Yawen L., Shaw R. (2019). Role of Civil Society in Sustainable Urban Renewal (Machizukuri) after the Kobe Earthquake. Sustainability.

[49] Paton D., Sagala S. (2018). Disaster Risk Reduction in Indonesia.

[50] Zhang S. (2007). Traditional Chinese philosophy of harmony of man with nature. Qiushi.

[51] Xiong D. (2010). Traditional Chinese Agricultural Culture.

[52] Australian Bureau of Meteorology (2008). About the Australian Tsunami Warning System Project. Commonwealth of Australia. www.bom.gov.au/tsunami/about_atws.shtml.

[53] Paton D., Johnston D., Rossiter K., Buergelt P., Richards A., Anderson S. (2017). Community understanding of tsunami risk and warnings in Australia. Aust. J. Emerg. Manag..

[54] Paton D., Frandsen M., Johnston D. (2010). Confronting an Unfamiliar Hazard: Tsunami preparedness in Tasmania. Aust. J. Emerg. Manag..

[55] Paton D., McClure J. (2013). Preparing for Disaster: Building Household and Community Capacity.

[56] Chen J.C., Chen C.T., Paton D., Buergelt P.T., McCaffrey S., Tedim F. (2015). Discourse on Taiwanese Forest Fires. Wildfire Hazards, Risks and Disasters.

[57] Onuma H., Shin K.J., Managi S. (2017). Household preparedness for natural disasters: Impact of disaster experience and implications for future disaster risks in Japan. Int. J. Disaster Risk Reduct..

[58] Paton D., Anderson E., Becker J., Peterson J. (2015). Developing a Comprehensive Model of Earthquake Preparedness: Lessons from the Christchurch earthquake. Int. J. Disaster Risk Reduct..

[59] Paton D., Kelly G., Bürgelt P.T., Doherty M. (2006). Preparing for Bushfires: Understanding intentions. Disaster Prev. Manag..

[60] Paton D., Smith L.M., Johnston D. (2000). Volcanic hazards: Risk Perception and Preparedness. New Zealand J. Psychol..

[61] Statistics New Zealand (2012). Census of Population and Dwellings: Table Builder. http://www.stats.govt.nz/tools_and_services/tools/TableBuilder/2006-census-pop-dwellings-tables/culture-and-identity/ethnic-group.aspx.

[62] McIvor D., Paton D., Johnston D.M. (2009). Modelling community preparation for natural hazards: Understanding hazard cognitions. J. Pac. Rim Psychol..

[63] Paton D. (2019). Disaster risk reduction: Psychological perspectives on preparedness. Aust. J. Psychol..

[64] Renn O. (2011). The social amplification/attenuation of risk framework: Application to climate change. Wiley Interdiscip. Rev. Clim. Chang..

[65] Paton D., Bürgelt P.T., Prior T. (2008). Living with Bushfire Risk: Social and environmental influences on preparedness. Aust. J. Emerg. Manag..

[66] Lion R., Meertens R.M., Bot I. (2002). Priorities in information desire about unknown risks. Risk Anal..

[67] Poortinga W., Pidgeon N.F. (2004). Trust, the asymmetry principle, and the role of prior beliefs. Risk Anal..

[68] Rippl S. (2002). Cultural theory and risk perception: A proposal for a better measurement. J. Risk Res..

[69] Dalton J.H., Elias M.J., Wandersman A. (2007). Community Psychology: Linking Individuals and Communities.

[70] Paton D., Buergelt P.T., Paton D., Tedim F. (2012). Community engagement and wildfire preparedness: The influence of community diversity. Wildfire and Community: Facilitating Preparedness and Resilience.

[71] Rink F.A., Jenn K.A., Crisp R.J. (2010). Divided we fall, or united we stand? How identity processes affect faultline perceptions and the functioning of diverse teams. The Psychology of Social and Cultural Diversity.

[72] Thatcher S.M., Jehn K.A., Zanutto E. (2003). Cracks in diversity research: The effects of diversity faultlines on conflict and performance. Group Decis. Negot..

[73] Lang D.J., Wiek A., Bergmann M., Stauffacher M., Martens P., Moll P., Swilling M., Thomas C.J. (2012). Transdisciplinary research in sustainability science: Practice, principles, and challenges. Sustain. Sci..

[74] Ismail-Zadeh A.T., Cutter S.L., Takeuchi K., Paton D. (2017). Forging a paradigm shift in disaster science. Nat. Hazards.

[75] Konno N., Nonaka I., Ogilvy J. (2014). Scenario Planning: The Basics. World Futures.

[76] Konno N., Nonaka I., Ogilvy J. (2014). The Mind of the Scenario Thinker. World Futures.

[77] Cameron L., Turner R.N., Crisp R.J. (2010). The application of diversity-based interventions to policy and practice. The Psychology of Social and Cultural Diversity.

[78] Paton D. (2008). Risk communication and natural hazard mitigation: How trust influences its effectiveness. Int. J. Glob. Environ. Issues.

[79] Curnin S., Owen C., Paton D., Trist C., Parsons D. (2015). Role clarity, swift trust and multi-agency coordination. J. Contingencies Crisis Manag..

